# Radiofrequency Microneedling for Facial Rejuvenation: A Systematic Review

**DOI:** 10.1111/jocd.70845

**Published:** 2026-04-07

**Authors:** Narendra Kumar, Dong Hye Suh, Sang Jun Lee, Shaheen Amna Kasif, Jean D. A. Carruthers

**Affiliations:** ^1^ Global Medical Affairs, Jeisys Medical Inc Seoul Korea; ^2^ ArumdauNara Dermatologic Clinic Seoul Korea; ^3^ Clinical Professor (Ophthalmology) University of British Columbia Vancouver British Columbia Canada

## Abstract

**Background:**

Radiofrequency microneedling (RFMN) is a prominent minimally invasive treatment for facial rejuvenation. While individual studies report positive outcomes, a comprehensive synthesis of evidence across efficacy, safety, and patient satisfaction is lacking.

**Objectives:**

To synthesize evidence on the aesthetic, safety, tolerability, and psychological outcomes of RFMN for facial rejuvenation.

**Methods:**

We conducted this systematic review in accordance with PRISMA and JBI guidelines. We systematically searched four electronic databases (PubMed, Embase, CENTRAL, LILACS) for studies published between 2015 and 2025. We included 22 studies. We assessed risk of bias using a JBI tool and conducted a thematic synthesis of aesthetic outcomes, patient satisfaction, and safety. We evaluated confidence in the findings using the GRADE‐CERQual approach.

**Results:**

Our synthesis of 20 studies (558 participants) found that RFMN treatments consistently improved aesthetic outcomes, with significant improvements in skin texture and tightening supported by high Global Aesthetic Improvement Scale scores. We found a favorable safety profile, where adverse events were predominantly mild and transient (erythema and edema were most common), and no serious complications were reported. Pain was typically mild to moderate and well‐tolerated. Patient satisfaction was remarkably high, with most studies reporting rates exceeding 90%, which was strongly correlated with the procedure's efficacy and minimal downtime.

**Conclusion:**

The synthesis of evidence indicates that RFMN is an effective and well‐tolerated intervention for facial rejuvenation, characterized by robust clinical outcomes and exceptionally high patient satisfaction. These findings solidify its role as a cornerstone treatment in modern aesthetic practice.

**Trial Registration:**

PROSPERO Registration number: CRD420251152380

## 1 Introduction

The field of facial rejuvenation has seen a significant shift towards minimally invasive procedures that offer discernible results with reduced patient downtime [1]. Among the array of available technologies, energy‐based devices represent a cornerstone of modern dermatologic practice. Specifically, radiofrequency (RF) technology has been established as a key therapeutic modality for minimizing the appearance of expression lines and addressing signs of skin aging by promoting collagen remodeling [1].

Radiofrequency microneedling (RFMN) represents a technological advancement that delivers monopolar & bipolar RF energy directly to the dermis via insulated and non‐insulated microneedles. This method creates controlled microthermal zones with minimal epidermal damage, thereby initiating a wound‐healing response that stimulates neocollagenesis and elastogenesis [1, 2]. Clinical studies have documented that both non‐ablative and ablative RF can rejuvenate and improve skin features across various skin types [1]. Concurrently, RFMN as a standalone treatment has gained popularity for facial skin aging, with recent systematic reviews demonstrating high patient satisfaction and a favorable safety profile [3].

Nevertheless, much of the existing literature on RFMN focuses predominantly on technical parameters and objective efficacy measures. A recent systematic review on radiofrequency, while confirming its efficacy, also highlighted a substantial discrepancies in the types and parameters used across studies [1, 2]. Similarly, a systematic review of microneedling pointed to a lack of standardized measures for esthetic outcomes [3]. In contrast, subjective outcomes, such as patient‐reported satisfaction, tolerability, perceived improvement, and expectations, remain underexplored and inconsistently reported. Given the inherently perceptual and satisfaction‐driven nature of cosmetic interventions, a comprehensive understanding of the patient's experience is essential. Therefore, this systematic review aims to address this gap by synthesizing evidence on patient satisfaction, treatment tolerability, and overall experience, while consolidating consistent findings on the objective efficacy and safety of RFMN for facial rejuvenation. The findings of this review will provide a consolidated evidence base to inform clinical practice, guide treatment protocols, and improve patient counseling.

## 2 Methods

The protocol for this systematic review was prospectively registered in PROSPERO (CRD420251152380). The review followed PRISMA 2020 guidelines for reporting systematic reviews.

### 2.1 Information Sources and Strategy

A systematic search was conducted in PubMed, Embase, Cochrane CENTRAL, and LILACS for studies published between January 1, 2015, and March 15, 2025. The search strategy combined MeSH/Emtree terms and free‐text keywords related to fractional microneedling, radiofrequency, and facial rejuvenation. The search was limited to English‐language publications. The complete search strategies for each database are provided in File S1 (Risk of Bias, Theme Synthesis, Themes, Search Strategies).

### 2.2 Study Selection and Screening

Three reviewers independently screened titles and abstracts. Subsequently, the full texts of all potentially eligible studies were assessed against the eligibility criteria, and all reasons for exclusion were documented. To safeguard against error, a fourth reviewer verified the exclusion decisions for the studies. No discrepancies were identified during this verification process.

### 2.3 Data Extraction

The lead reviewer performed the initial data extraction into a standardized template. To ensure accuracy, a second reviewer then cross‐checked the extracted data from all studies. The two reviewers discussed any instances of disagreement until they reached a consensus.

### 2.4 Quality Appraisal (Risk of Bias)

The methodological quality of included studies was assessed by three reviewers using the Joanna Briggs Institute (JBI) Critical Appraisal Checklist appropriate for each study design. Specifically, we used the JBI checklist for randomized controlled trials (*n* = 4), the JBI checklist for prospective studies (cohort and prospective designs; *n* = 7), and the JBI checklist for quasi‐experimental studies (case series and non‐comparative studies; *n* = 9). To ensure consistency and minimize bias, the reviewers applied the checklists in a standardized, pre‐piloted manner, and all appraisal decisions were subsequently reviewed and confirmed by the senior author. The complete risk of bias assessments are provided in the File S1.

### 2.5 Data Synthesis

We synthesized the findings using a thematic analysis approach. Findings were analyzed across three pre‐specified domains [1]. Aesthetic Outcomes [2], Satisfaction and Psychological Impact, and [3]. Safety and Tolerability. Due to significant heterogeneity, a narrative synthesis was conducted. Given the heterogeneity of study designs and outcome measures, a meta‐analysis was not feasible. Therefore, we assessed the certainty of the evidence for each key finding using a narrative approach following established guidance for systematic reviews without meta‐analysis. For each finding, we considered the study design, risk of bias from the JBI assessments, consistency of findings across studies, the directness of the evidence to the review question, and, where applicable, the precision of the estimates. Based on these factors, we assigned each finding a certainty rating of high, moderate, low, or very low. These assessments are presented alongside each key finding in the Results section.

### 2.6 Assessment of Heterogeneity

We assessed heterogeneity among the included studies qualitatively, considering variability in RFMN technologies, treatment parameters, outcome measures, and participant characteristics. This high degree of heterogeneity informed the decision to conduct a narrative synthesis, as a meta‐analysis was deemed infeasible.

## 3 Results and Synthesis

### 3.1 Study Selection

**FIGURE 1:**
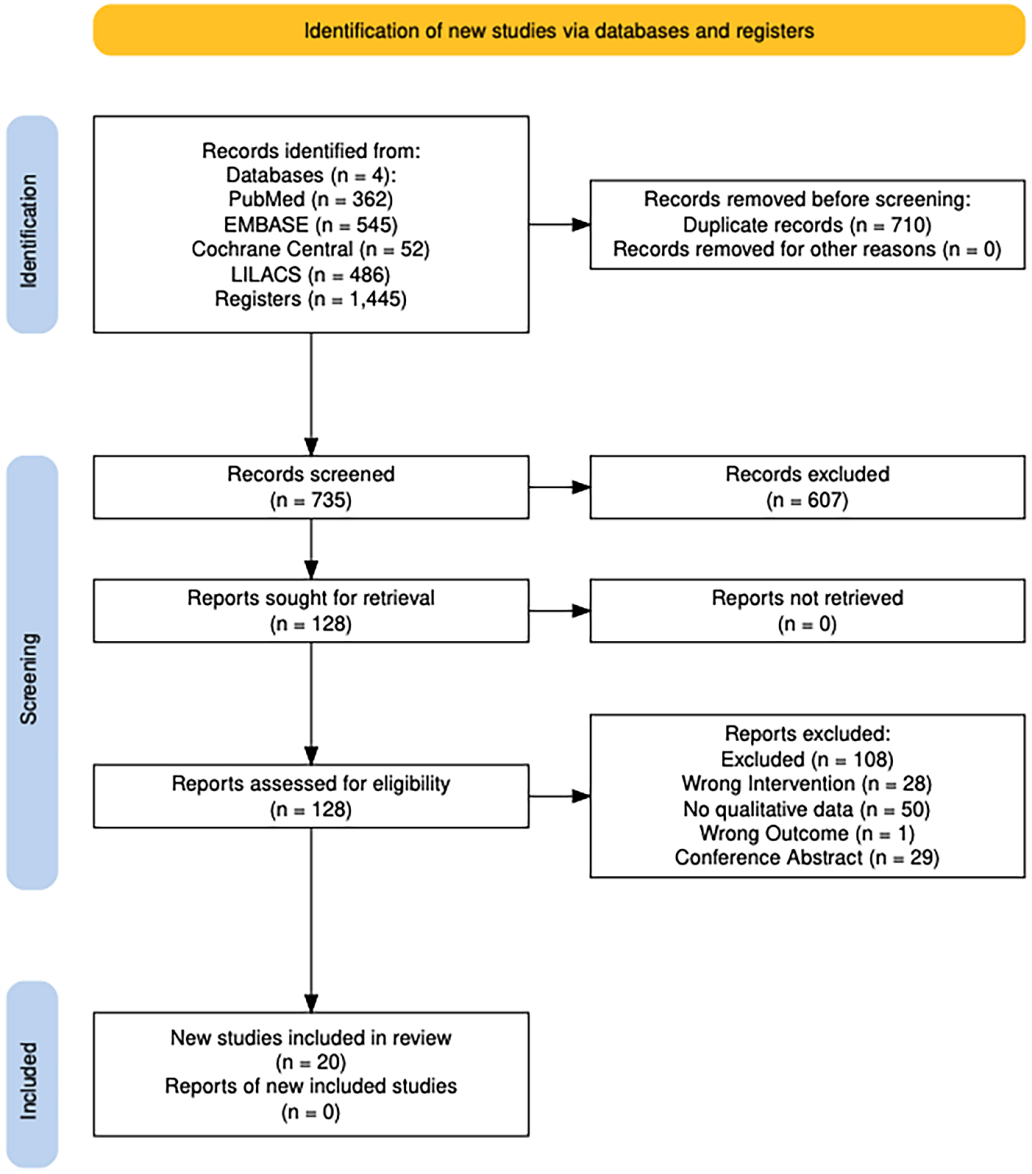
PRISMA 2020 flowchart illustrates the identification, screening, eligibility, and inclusion of studies. Numbers correspond to records at each phase (*n* = 1445 initial records; *n* = 20 included).

A total of 1445 articles were identified through database searching. After removing duplicates and applying inclusion criteria, 20 articles were included in the final synthesis (see PRISMA diagram, Figure 1).

### 3.2 Characteristics of Included Studies

The 20 studies included in this review, published from 2015 to 2025, collectively involved 558 patients, [4, 5, 6, 7, 8, 9, 10, 11, 12, 13, 14, 15, 16, 17, 18, 19, 20, 21, 22, 23]. Geographically, the evidence base was dominated by research from Asia (*n* = 13) [4, 6, 9, 10, 11, 13, 14, 16, 17, 20, 21, 22, 23], North America (*n* = 3) [7, 8, 12], and Europe (*n* = 2) [5, 19]. Two studies did not report their location [15, 18]. The most common study design was the case series (*n* = 8) [6, 7, 8, 10, 11, 20, 21, 22], while prospective studies (*n* = 8) [5, 9, 12, 14, 16, 18, 23], and randomized controlled trials were less frequent (*n* = 4) [4, 13, 15, 17].

Sample sizes ranged from 9 to 133 participants, with a mean study size of approximately 32 patients [4, 5, 6, 7, 8, 9, 10, 11, 12, 13, 14, 15, 16, 17, 18, 19, 20, 21, 22, 23]. The cohort was predominantly female (*n* = 531), with 27 male participants. The mean age of patients across studies was 49.8 years, with a range from 38 to 85 years [4, 5, 6, 7, 8, 9, 10, 11, 12, 13, 14, 15, 16, 17, 18, 19, 20, 21, 22, 23]. Indications for treatment were primarily facial and neck rejuvenation, targeting skin laxity, rhytids, and textural irregularities [4, 5, 6, 7, 8, 9, 10, 11, 12, 13, 14, 15, 16, 17, 18, 19, 20, 21, 22, 23].

A variety of RFMN devices were investigated. The most used systems in the studies were Lutronic (Genius/INFINI, *n* = 6) [5, 7, 9, 16, 17, 19], and EndyMed Medical (Intensif/3DEEP, *n* = 3) [6, 12, 20]. Other devices included models from InMode [8], Jeisys Medical [11, 14], Peninsula Medical [4, 10], and Viol Co [23]. Treatment protocols varied. Still, most studies administered one to three treatment sessions at intervals of three to four weeks [4, 11, 12, 13, 14, 16, 17, 19, 22, 23]. Energy delivery depths typically ranged from 0.5 mm to 3.5 mm, targeting anatomical areas including the whole face, periorbital region, lower face, jawline, and neck. A summary of the characteristics of the included studies is provided in Table 1.

**TABLE 1:** Characteristics of studies included in the systematic review (*n* = 20).

First author (Year)	Country	Study design	Participants (*n*, F/M)	RFMN device	Key outcomes measured
Liu (2024) [4]	China	RCT	18 (17/1)	Gold Microneedle United II	Skin tightening, wrinkles, satisfaction, safety
Nguyen (2022) [5]	Germany	Prospective	29 (29/0)	Genius (Lutronic)	Skin tightening, GAIS, satisfaction, safety
Xiao (2021) [6]	China	Case series	98 (98/0)	Intensif (EndyMed)	Neck aging, satisfaction, safety
Gold (2020) [7]	USA	Case series	12 (12/0)	Genius (Lutronic)	Skin texture, wrinkles, GAIS, satisfaction
Demesh (2020) [8]	USA	Case series	9 (9/0)	Morpheus8 (InMode)	Skin laxity, clinician assessment, safety
Tanaka (2017) [9]	Japan	Prospective	15 (14/1)	INFINI (Lutronic)	Skin tightening, satisfaction, safety
Lin (2025) [10]	China	Case series	18 (18/0)	MicroRF9 (Peninsula)	Periorbital wrinkles, GAIS, FWES, safety
Zhang (2018) [11]	China	Case series	27 (26/1)	INTRAcel (Jeisys)	Photoaging, skin tightening, satisfaction
Elawar (2018) [12]	USA	Prospective	19 (13/6)	3DEEP (EndyMed)	Acne scars, pores, texture, GAIS, safety
Lu (2017) [13]	China	RCT	13 (13/0)	Bodytite (Invasix)	Skin laxity, GAIS, safety
Lee (2015) [14]	South Korea	Prospective	20 (19/1)	INTRAcel (Jeisys)	Periorbital wrinkles, satisfaction, safety
Kauvar (2022) [15]	Israel	RCT	9 (9/0)	Novel Device	Wrinkles, texture, GAIS, histology
Park (2021) [16]	South Korea	Prospective	25 (23/2)	GENIUS (Lutronic)	Wrinkles, GAIS, satisfaction, safety
Dou (2021) [17]	China	RCT	15 (13/2)	INFINI (Lutronic)	Baggy eyelids, satisfaction, safety
Kaplan (2016) [18]	Israel	Prospective	14 (13/1)	EndyMed PRO	Skin rejuvenation, satisfaction, safety
Clementoni (2016) [19]	Italy, USA	Prospective	33 (26/7)	INFINI (Lutronic)	Skin laxity, craniometry, and satisfaction
Tanaka (2015) [20]	Japan	Case series	20 (19/1)	Intensif (EndyMed)	Skin tightening, 3D volume, satisfaction
Hong (2025) [21]	South Korea	Case series	12 (11/1)	DoubleTite (AGNES)	Neck rejuvenation, GAIS, safety
Baek (2023) [22]	South Korea	Case series	19 (19/0)	Corage 2.0 (Quanteq)	Wrinkles, skin elasticity, and safety
Serdar (2019) [23]	Turkey	Prospective	133 (130/3)	Scarlet S (Viol)	Wrinkles, satisfaction, safety

### 3.3 Methodological Quality and Risk of Bias of Included Studies

The quality assessment of the included studies showed that the risk of bias was closely tied to the study design. Most studies had clear goals and used appropriate methods. However, we identified consistent patterns of limitations. A lack of blinding primarily compromised the four randomized controlled trials. The eight prospective cohort studies often failed to account for or control for confounding factors. While the eight case series were frequently rated as having a low risk of bias according to their specific checklist, this assessment is constrained by their inherent lack of a control group. The checklist was independently applied to all 20 included studies. Complete results of the risk of bias assessment are summarized in the File S1. This pattern reflects the expected methodological spectrum of clinical evidence in aesthetic medicine, where practical constraints often limit the feasibility of randomized designs.

### 3.4 Aesthetic Outcomes

Across the included studies, improvements in perceived aesthetic outcomes were widely reported. A thematic synthesis of clinician‐ and patient‐reported outcomes identified three main subthemes, described below (Table 2). Based on our narrative assessment of certainty, considering inconsistent findings across multiple study designs, a generally low to moderate risk of bias, and the directedness of outcomes, we have high certainty in the evidence supporting aesthetic improvement.

**TABLE 2:** Summary of key findings by category.

Theme	Key finding	Magnitude of effect (Range)	Supporting studies	Total patients (*n*)
Aesthetic outcomes				
	Improvement in rejuvenation	> 90% of patients showing improvement	10/20	(*n* = 382) [4, 6, 7, 11, 15, 16, 17, 19, 21, 23]
	Improvement in skin texture	Significant improvement in GAIS and instrumental measures	7/20	(*n* = 147) [5, 7, 10, 11, 12, 15, 19]
	Lifting and tightening effect	Visible tightening and significant craniometric change	5/20	(*n* = 109) [4, 5, 8, 19, 20]
Safety and tolerability				
	Incidence of transient erythema/edema	Very common, resolving within hours to days	11/20	(*n* = 405) [4, 5, 6, 8, 9, 11, 14, 16, 21, 22, 23]
	Mean pain score (0–10 VAS)	3.4; range from mild to moderate	8/20	(*n* = 156) [4, 5, 8, 9, 10, 19, 20]
Satisfaction				
	Patient satisfaction rate	82% to 100% of patients	17/20	(*n* = 518) [4, 5, 6, 9, 10, 11, 12, 13, 14, 15, 16, 17, 18, 19, 20, 21, 23]
	Willingness to recommend procedure	94% to 100% of patients	3/20	(*n* = 56) [9, 14, 21]

*Note:* Data is presented as the range of results reported across the included studies. The denominator for contributing studies is the total number of included studies (*n* = 20).

### 3.5 Rejuvenation

Most studies documented comprehensive rejuvenation effects, including the reduction of rhytids and improvement in overall skin quality [4, 6, 7, 11, 15, 16, 17, 19, 21, 23]. Improvements in wrinkles and skin tightening were highly prevalent, with over 90% of patients showing positive results in several analyses [11]. This was consistently reflected in clinician assessments, where a significant majority of patients were rated as “improved” to very much improved on the Global Aesthetic Improvement Scale (GAIS) [7, 12]. Objective measures, such as 3D volumetric analysis, confirmed sustained tissue tightening and contour improvement for up to six months [20]. The synergistic effect of combining RFMN with other modalities, such as a fractional thulium laser, was noted to provide superior rejuvenation compared to RFMN alone [16].

### 3.6 Skin Texture Improvement

Enhancements in skin texture, including reductions of pore size and improvements in surface irregularities, have been documented across multiple studies [5, 7, 10, 11, 12, 15, 19]. Investigator assessments on the GAIS indicated significant progress, with mean scores corresponding to an ‘improved’ to ‘much improved’ appearance at the 90‐day follow‐up [5]. Both blinded evaluators and patients noted significant improvements in skin texture, with one study reporting that 100% of participants perceived improvement at 3 months post‐treatment [15]. These findings were supported by instrumental measurements, such as the Fitzpatrick Wrinkle and Elastosis Scale (FWES) and Antera 3D imaging, which showed significant reductions in wrinkle depth and volume, particularly in the periorbital area [10].

### 3.7 Lifting and Tightening

A specific tightening effect, especially on the lower face, jawline, and neck, was a prominent finding [4, 5, 8, 19, 20]. Clinical observations noted visible skin tightening in the submental and jawline regions [5]. This was particularly noted in the treatment of laxity following facialplasty, where RFMN improved jowl and neck contour [8]. The lifting effect was also quantifiable, with one study demonstrating a significant decrease in the cervicomental and gnathion angles, indicating a more defined jawline and reduced neck laxity [19].

### 3.8 Safety, Pain, and Tolerability

The analysis of safety, pain, and tolerability across the included studies identified three consistent subthemes: the nature of adverse events, the experience of pain during treatment, and the overall tolerability of the procedures. Based on our narrative assessment of certainty, considering the consistent findings across studies, direct patient‐reported outcomes, and the predictable pattern of adverse events, we have high certainty in the evidence supporting a favorable safety and tolerability profile.

### 3.9 Adverse Events

The reported adverse events were uniformly characterized as mild and transient. The most common events were erythema and edema, which were frequently reported across studies and typically resolved within hours to a few days without intervention [5, 6, 8, 9, 11, 14, 16, 21, 22]. Other minor events included purpura [4], petechiae, and crusting [6], and transient numbness [8]. One study reported a single serious adverse event (pharyngeal inflammation) that resolved within 2 days [5]. Crucially, no studies reported occurrences of scarring or severe infections [4, 5, 11, 14]. Furthermore, in the few cases where post‐inflammatory hyperpigmentation was reported, it was transient and resolved completely [14]. In studies that directly compared RFMN to other laser modalities, the adverse event profile was favorable, with a low incidence of post‐inflammatory hyperpigmentation [14, 23].

### 3.10 Pain During Treatment

The experience of pain during RFMN procedures varied across studies and anatomical treatment areas. One study that quantified pain using a 10‐point Visual Analog Scale (VAS) reported an average score of 3.4, indicating mild discomfort [4]. This finding of generally mild pain was supported by several other studies that described the treatment as “well tolerated,” citing “minimal discomfort” even without anesthesia or cooling [8, 9, 20] However, other studies provided more detailed data, revealing that a significant number of patients experienced moderate pain. One study reported that 72% of patients described moderate pain, with 7% reporting significant pain [18]. Similarly, another study found that 33.3% of patients experienced moderate pain and 22.2% severe pain when treating the sensitive periorbital area [10]. Another study corroborated this, noting that all patients reported mild to moderate pain during treatment [19]. Despite this variability, the pain was consistently noted as transient and self‐limiting, subsiding shortly after the procedure concluded [10, 19].

### 3.11 Treatment Tolerability

Overall, RFMN treatments were well‐tolerated by patients. The combination of transient, mild adverse events and manageable pain levels contributed to a favorable tolerability profile. This is evidenced by high rates of treatment completion across all studies and the fact that all reported adverse events were resolved spontaneously without sequelae [4, 6, 8, 11, 14, 16, 21]. The rapid resolution of side effects, often within 48 h, was a key factor in the reported patient satisfaction and the procedure's characterization as having minimal downtime [5, 6, 20].

The certainty of evidence for each key finding, assessed using the GRADE‐CERQual approach, is summarized in Table 3.

**TABLE 3:** Qualitative findings and CERQual assessments.

Review finding	Methodological limitations	Consistency	Adequacy of data	Relevance	Certainty rating	Explanation of certainty rating
Improvement in rejuvenation	Minor concerns	High	Adequate	Directly relevant	High	Consistent reports across 11 studies, with > 90% of patients showing improvement in rhytids and skin quality, supported by clinician assessments (GAIS) and objective measures.
Improvement in skin texture	Minor concerns	High	Adequate	Directly relevant	High	Significant improvement documented across 8 studies, including 100% patient‐perceived improvement in one study and corroboration with instrumental measurements (e.g., FWES, Antera 3D).
Lifting and tightening effect	Moderate concerns	High	Adequate	Directly relevant	Moderate	A specific tightening effect was consistently reported across 5 studies, supported by clinical observation and quantifiable craniometric changes, but based on a smaller number of studies.
High patient satisfaction and willingness to recommend	Minor concerns	High	Rich	Directly relevant	High	Based on a large volume of data from 18 studies, showing consistent satisfaction rates exceeding 90% and a high willingness to recommend.
Favorable safety profile	Minor concerns	High	Adequate	Directly relevant	High	A predictable profile of mild, transient erythema and edema was uniformly reported across 11 studies, with no serious complications.
Pain is manageable and transient	Minor concerns	High	Adequate	Directly relevant	High	Pain was consistently reported as variable (mild to moderate) but manageable across 7 studies, with low VAS scores and transient duration.
Correlation with clinician assessment	Minor concerns	High	Adequate	Directly relevant	Moderate	A positive relationship was observed in studies measuring both, but this finding is based on a more limited subset of the evidence.

*Note:* Certainty ratings are defined as High, Moderate, Low, or Very Low.

## 4 Discussion

This systematic review evaluated the aesthetic outcomes, safety, and patient satisfaction associated with fractional microneedling radiofrequency (RFMN) treatment for facial rejuvenation. The analysis of 20 studies demonstrates that RFMN is an effective intervention characterized by high patient satisfaction, which appears to be intrinsically linked to its favorable safety and tolerability profile.

### 4.1 The Satisfaction‐Safety Profile

This review demonstrates that RFMN's high efficacy is complemented by an exceptionally favorable safety profile, suggesting a positive benefit–risk profile for patients and clinicians. This balance is reflected in remarkably consistent patient satisfaction, with most studies documenting rates exceeding 90% [9, 11, 19], with some reporting perfect (100%) satisfaction rates at follow‐up periods of up to 12 months [9, 19]. However, it should be noted that most studies reported outcomes up to 6 months, with only three studies providing data beyond 1 year [9, 13, 19], and limited evidence beyond this timeframe.

The clinical relevance of this finding is underscored when these satisfaction rates are contextualized with the concurrent safety and tolerability data. Patient satisfaction in aesthetic medicine is not solely dependent on efficacy but is profoundly influenced by the treatment experience and the burden of adverse events.

In this review, high satisfaction is associated with a consistently reported profile of mild, transient adverse events. The most common side effects, such as erythema and edema, were uniformly short‐lived, typically resolving within hours to a few days without intervention [5, 6, 8]. Crucially, no studies reported serious, long‐term complications such as scarring or permanent pigmentary changes [4, 5, 11, 14].

Furthermore, while the experience of pain was variable and could be moderate in more sensitive areas, such as the periorbital region [10, 18] it was consistently described as manageable, transient, and subsiding quickly after the procedure [10, 19]. The fact that treatments were characterized as “well tolerated” with “minimal downtime” [5, 6, 20], suggests that the overall patient experience is positive. This indicates that RFMN delivers clinically demonstrable aesthetic improvements, such as skin tightening, texture enhancement, and wrinkle reduction, with minimal disruption to the patient's daily life. The high satisfaction, therefore, reflects a favorable balance between significant cosmetic efficacy and an exceptionally manageable recovery process, both of which are essential for individuals seeking noticeable results with minimal social downtime.

The collective evidence from this systematic review solidifies Fractional Microneedling Radiofrequency (RFMN) as a cornerstone treatment in facial rejuvenation, characterized by a uniquely favorable balance of high efficacy, a positive patient experience, and a strong safety profile. The high rates of patient satisfaction and willingness to recommend the procedure, as documented across numerous studies, [11, 12, 19] are not isolated findings but are directly supported by the objective clinical outcomes and minimal downtime reported in the literature [5, 9]. This positions RFMN as a particularly compelling option when compared with other standard energy‐based devices.

A key differentiator for RFMN is its lower treatment burden and greater tolerability compared with laser modalities. For instance, a recent split‐face study comparing two fractional Nd: YAG lasers for photoaging required five treatment sessions and reported significantly higher pain levels with the picosecond laser [24]. This stands in sharp contrast to the findings of the present review, where RFMN demonstrated a comparable high‐efficacy profile for skin rejuvenation [4, 7] but was consistently described as “well tolerated” with “minimal discomfort” [8, 20]. The lower pain experience and potentially fewer required sessions contribute significantly to the high patient satisfaction documented in our review.

An important consideration when interpreting these findings is the substantial heterogeneity across included studies. Variability in RFMN devices, energy settings, needle depths, number of sessions, and treated facial areas limits direct comparability between studies and constrains the generalizability of results to specific protocols or clinical contexts. While our narrative synthesis captures the consistent direction of effect across this heterogeneous evidence base, the optimal treatment parameters and their comparative effectiveness remain undefined. Future research should prioritize standardized outcome measures and head‐to‐head comparisons of different devices and protocols to enable more precise clinical recommendations.

Mechanistically, RFMN's safety, especially for diverse skin types, is rooted in its epidermal‐sparing action. As detailed in a recent review, [25]. RFMN delivers thermal energy directly to the dermis, creating sublative injury zones that stimulate neocollagenesis while preserving the overlying epidermis. This principle directly explains the low rate of serious side effects and transient adverse events, such as erythema and edema, which resolved within days [5, 14, 20]. This makes RFMN a strategically safer option for patients with darker skin phototypes (Fitzpatrick III‐VI), for whom procedures causing epidermal disruption carry a heightened risk of dyspigmentation, [25] a finding corroborated by the low incidence of post‐inflammatory hyperpigmentation in our review [23].

Furthermore, the value of RFMN builds on the established benefits of basic microneedling. A foundational systematic review [2] concluded that microneedling is preferred due to minimal side effects and shorter downtime and noted its enhanced potential when combined with radiofrequency. The findings of the present review confirm and extend this conclusion, demonstrating that these inherent benefits are successfully leveraged and enhanced. The high rates of “improved” or “much improved” GAIS scores [6, 21], and significant objective measures such as sustained tissue tightening [20] indicate that RFMN provides superior and durable aesthetic outcomes.

It is important to mention the recent FDA advisory on potential risks with certain uses of RFMN. In our reviewed studies, the needle depth was largely intradermal, and we did not observe any fat necrosis [26]. RFMN is a medical procedure, and it should be performed only by a trained healthcare professional.

### 4.2 Publication Bias and Selective Reporting

A formal assessment of publication bias was not performed due to heterogeneous reporting of outcomes. However, our search identified unpublished studies in trial registries that could not be included due to insufficient data. The published literature shows a clear predominance of positive results with minimal documentation of null findings. This pattern, combined with the fact that all quasi‐experimental studies (nearly half the evidence) reported positive outcomes, suggests likely publication bias. The consistent positive results in this review should therefore be interpreted with this limitation in mind.

## 5 Limited Follow‐Up Duration

An additional limitation concerns the duration of follow‐up across the included studies. Follow‐up periods ranged from 1 to 12 months post‐treatment, with most studies reporting outcomes at 3 to 6 months. Only three studies [9, 13, 19], provided data at or beyond 1 year, and none exceeded 12 months. Consequently, the durability of aesthetic improvements and long‐term patient satisfaction beyond this timeframe remains uncertain. While the available evidence suggests sustained effects for 6 to 12 months, future studies with extended follow‐up periods are needed to confirm the longevity of RFMN treatment effects and to assess whether satisfaction is maintained over several years.

### 5.1 Sensitivity Analysis

A quantitative sensitivity analysis was not possible. Instead, we qualitatively assessed how study quality influenced our results. The main findings about high satisfaction, effectiveness, and safety stayed consistent across studies with varying risk‐of‐bias ratings. These results were supported by both cohort studies (moderate risk) and quasi‐experimental studies (low risk), and they aligned with the findings from the RCTs, despite the RCTs' higher risk of bias. This consistency across different methodological approaches increases confidence in our primary conclusions.

## 6 Conclusion

In conclusion, this review confirms Radiofrequency Microneedling (RFMN) as an important dermatological treatment that effectively combines minimal downtime with good results. By encouraging dermal remodeling with minimal epidermal damage, RFMN delivers patient‐pleasing outcomes with a very safe profile, strengthening its vital role in modern aesthetic practice.

## Author Contributions

All authors contributed equally to the conceptualization, methodology, search strategy, study selection, screening, data synthesis, interpretation, and preparation of the original draft.

## Ethics Statement

Ethics approval was not required for this study as it was a systematic review of previously published literature and did not involve direct interaction with human subjects.

## Conflicts of Interest

Dr. Dong Hye Suh is a speaker for Jeisys Medical Inc. Dr. Narendra Kumar, and Dr. Shaheen Amna Kasif works at Global Medical Affairs, Jeisys Medical Inc. Dr. San Jun Lee and Dr. Jean Carruthers declare no conflicts of interest.

## Supporting information


**File S1:** Is an Excel file containing the complete search strategies, risk‐of‐bias assessments (JBI checklists), data extraction tables, full‐text screening log, and theme synthesis data.

## Data Availability

The data that support the findings of this study are available on request from the corresponding author.
